# Non-invasive assessment of adrenocortical activity as a measure of stress in giraffe (*Giraffa camelopardalis*)

**DOI:** 10.1186/s12917-016-0864-8

**Published:** 2016-10-18

**Authors:** Meredith J. Bashaw, Florian Sicks, Rupert Palme, Franz Schwarzenberger, Adrian S. W. Tordiffe, Andre Ganswindt

**Affiliations:** 1 Wildlife Reproductive Centre, Taronga Conservation Society of Australia, Dubbo, NSW Australia; 2 Department of Psychology, Franklin & Marshall College, Lancaster, PA USA; 3 Tierpark Berlin, Berlin, Germany; 4 Department of Biomedical Sciences, University of Veterinary Medicine, Vienna, Austria; 5 Department of Paraclinical Sciences, Faculty of Veterinary Science, University of Pretoria, Onderstepoort, Pretoria, South Africa; 6 National Zoological Gardens of South Africa, Pretoria, South Africa; 7 Department of Anatomy and Physiology, Endocrine Research Laboratory, Faculty of Veterinary Science, University of Pretoria, Onderstepoort, Pretoria, South Africa

**Keywords:** ACTH challenge, Validation, Ungulate, Ruminant, Zoo, Endocrine, Adrenal physiology, Glucocorticoid, Health

## Abstract

**Background:**

Numbers of giraffes are declining rapidly in their native habitat. As giraffe research and conservation efforts increase, the demand for more complete measures of the impact of conservation interventions and the effects of captive environments on animal health and welfare have risen. We compared the ability of six different enzyme immunoassays to quantify changes in fecal glucocorticoid metabolites (FGM) resulting from three sources: adrenocorticotropic hormone stimulation test, transport, and time of day that samples were collected.

**Results:**

Two male giraffes underwent ACTH injections; all six assays detected FGM increases following injection for Giraffe 1, while only three assays detected FGM increases following injection for Giraffe 2. Consistent with other ruminant species, the two 11-oxoetiocholanolone assays (one for 11,17-dioxoandrostanes and the other for 3α,11-oxo metabolites) measured the most pronounced and prolonged elevation of FGM, while an assay for 3β,11β-diol detected peaks of smaller magnitude and duration. Both of the 11-oxoetiocholanolone assays detected significant FGM increases after transport in Giraffes 3–7, and preliminary data suggest FGM detected by the assay for 11,17-dioxoandrostanes may differ across time of day.

**Conclusions:**

We conclude the assay for 11,17-dioxoandrostanes is the most sensitive assay tested for FGM in giraffes and the assay for FGM with a 5β-3α-ol-11-one structure is also effective. 11-oxoetiocholanolone enzyme immunoassays have now been demonstrated to be successful in a wide variety of ruminant species, providing indirect evidence that 5β-reduction may be a common metabolic pathway for glucocorticoids in ruminants. As FGM peaks were detected in at least some giraffes using all assays tested, giraffes appear to excrete a wide variety of different FGM. The assays validated here will provide a valuable tool for research on the health, welfare, and conservation of giraffes.

**Electronic supplementary material:**

The online version of this article (doi:10.1186/s12917-016-0864-8) contains supplementary material, which is available to authorized users.

## Background

Giraffes (*Giraffa camelopardalis*) are currently listed as a species of least concern by the International Union for Conservation of Nature (IUCN) [1]. However, wild populations have recently suffered major declines, from an estimated 140,000 individuals to an estimated 80,000 individuals over the course of a decade, and two subspecies (*G. c. peralta* [2] and *G. c. rothschildi* [3]) are now recognized as endangered by IUCN. Against this backdrop, efforts to study and conserve wild giraffes and to ensure optimal health and welfare for captive giraffes have intensified.

Studies of wild populations are revealing that giraffes have complex social structures and habitat dynamics with implications for conservation strategies [4–7]. Giraffes’ habitat preferences are shaped by energetically costly reproductive strategies, resulting in sex segregation and female habitat preferences that change during lactation [8, 9]. Zoos maintain a population of captive giraffes that could be used as a hedge against extinction, but at the turn of the century some captive giraffes exhibited poor health [10–12] and behavioral problems [13–15]. Zoos have worked successfully to improve giraffe health and reduce abnormal repetitive behaviors [15–17], but these problems still persist at low rates so it is unclear whether captive environments are yet optimal for housing giraffes. The adrenal system plays a role in energy regulation [18], reproduction [19], immune function [20], and physiological responses to disturbance [21], so an efficient and ideally noninvasive measure of adrenal responses in giraffes could shed important light on population dynamics, conservation efforts, health, and welfare in both *in* and *ex situ* populations. To the best of our knowledge no data are currently available on hypothalamic-pituitary-adrenal (HPA) axis functioning in giraffes.

Measuring an individual’s physiological arousal provides a window into how that individual animal is coping with its environment, whether in the wild or captivity. As Webster [22] describes, arousal produced by exciting or stressful stimuli results in an activation of the HPA axis, which results in the release of glucocorticoid hormones (GC), amongst others. GC produce a negative feedback such that once the animal is no longer excited or stressed, the HPA axis response ends and GC return to baseline levels. If the organism is unable to resolve or escape the situation, HPA axis activation continues and negative feedback systems are disrupted, resulting in prolonged elevations of GC concentrations with negative consequences for the animals’ behaviour, health, and ability to respond to future events [23, 24]. In addition to within-animal changes, differences in GC levels between animals have been linked to behavioral differences that may contribute to or reflect different life history strategies [25, 26].

Fecal glucocorticoid metabolites (FGM) have become a popular measure of physiological function and welfare in a variety of species [27, 28] because they reflect adrenocortical activity over a certain time period [29] and can therefore be used to answer a wide variety of research questions. Technically, animals are not disturbed during fecal sample collection, and sampling is therefore feedback-free due to the absence of capture and handling. This is particularly beneficial for field studies, where capture and handling may be logistically challenging or cause long-term health consequences [30]. However, species-specific differences in the composition of excreted FGM and the different affinities of the antibodies used in each assay for specific FGM [31–33] require the measurement of FGM to be validated for each species [21, 29] and ideally each sex [34]. Validation is accomplished by experimentally stimulating the HPA axis using e.g., an injection of adrenocorticotrophic hormone (an ACTH challenge) or by subjecting the animal to a putative stressor (e.g., transport or aggressive interaction). These two validation techniques reveal two aspects of measurement effectiveness. Because an ACTH challenge provides direct physiological stimulation of the adrenal glands, it measures how well an assay detects an increase in GC concentrations. Subjecting an animal to a putative stressor, on the other hand, targets the degree to which the assay is sensitive enough to detect biologically-relevant changes in GC. If an assay detects increased FGM levels following the event, one can conclude both that the event was indeed stressful and that the assay is sensitive enough to detect an environmental stress of that magnitude. When using feces as a hormone matrix, these techniques allow researchers to verify that FGM measured with a particular assay reflect both increased GC concentrations and perceived stress [29].

This study aimed to demonstrate that measurement of FGM in giraffes provides a feasible and noninvasive way to assess the physiological function and affective state of individual giraffes. More specifically, this study: a) compared the ability of six different enzyme immunoassays (EIAs) to quantify changes in FGM produced by an adrenocorticotropic hormone stimulation test (ACTH challenge test) at two different sampling rates, b) measured changes in FGM concentrations in daily samples as a result of transport, a putative stressor, and c) preliminarily investigated the influence of time of day samples were collected on FGM results.

## Methods

### Study animals

A total of 7 giraffes were monitored in this study. Giraffe 1 was a 6-year old male South African (*Giraffa camelopardalis giraffa*) singly housed in a 4000 m^2^ enclosure at the National Zoological Gardens of South Africa (NZG), Pretoria, South Africa. Giraffe 2 was a 17-year-old male hybrid-subspecies *G. camelopardalis* housed in a 10-ha exhibit with two other adult male giraffes and zebra (*Equus burchellii*), eland (*Taurotragus oryx*), forest buffalo (*Syncerus caffer nanus*), and ostrich (*Struthio camelus*) at Taronga Western Plains Zoo, Dubbo, NSW, Australia. Giraffes 3–7 were a juvenile male (3), two juvenile females (4 and 5), and two adult females (6 and 7) transported among seven German zoos (see Table 1). The study of Giraffes 3–7 consisted solely of opportunistic non-invasive collection of feces during and after transfers that were occurring for management reasons.

**Table 1:** Demographic and transport information for focal animals 3–7

Giraffe	Sex	Subspecies	Age at Transport (years)	Transport Type	Transport Duration^a^ (h)	Samples Collected^b^	No. of Samples
3	M	*G. c. rothschildi*	1	Within-zoo	0.33	−10 to +30 d	*n* = 38
4	F	*G. c. reticulata*	3	Between-zoo	4	−38 to +41 d	*n* = 37
5	F	*G. c. reticulata*	2.5	Between-zoo	4.5	−3 to +14 d	*n* = 18
6	F	*G. c. angolensis*	10	Between-zoo	6	−8 to +38 d	*n* = 38
7	F	*G. c. angolensis*	11	Between-zoo	6	−5 to +38 d	*n* = 38

^a^The reported transport duration is the amount of time the animal was actually moving in the transport vehicle; the time to load, secure and unload the animal is not included

^b^Date range for sample collection relative to the date of transport, followed by number of samples collected. - indicates the number of days before transport and + indicates the number of days after transport

### ACTH challenge tests

Giraffe 1 was remotely injected into the caudal thigh muscle with Synacthen Depot (Novartis) loaded into a 10 ml dart syringe with a 60 mm × 2 mm standard needle (Dan-Inject, Denmark) at an estimated dose 1 IU/kg at 18:00 on day 1 (21 October 2012). The animal was continuously monitored and a fecal sample (3–4 pellets) collected from each defecation beginning 12 h before the ACTH injection and continuing until 72 h post-injection. Fecal samples were frozen within 1 h of collection.

Giraffe 2 was injected with approximately 0.7 IU/kg of Synacthen Depot using two darts delivered 2 min. apart into the shoulder beginning at 12:18 on day 6 (21 May 2013). To facilitate identification of the feces, the animal was fed glitter beginning 1 week before the injection. Fecal samples were collected daily between 08:00 and 10:00 for 5 days before and 4 days after injection and transferred to a −20 °C freezer within 30 min. Fecal samples from each defecation between 20 and 28 h post-injection were also collected; these samples were placed on ice after collection and transferred to a −20 °C freezer when animal care staff were available to let the researcher out of the exhibit. All samples were frozen within 1 h of defecation except two: one was on ice for 64 min and a second for 110 min before freezing.

### Transport

Giraffes 3–7 were monitored opportunistically during management-necessitated transfers between zoos or between exhibits within a zoo. Transports occurred in winter (Giraffes 3 and 5), summer (Giraffe 4), and autumn (Giraffes 6 and 7). For each transport, giraffes were loaded onto a vehicle, driven for at least 20 min (individual transport lengths given in Table 1), and unloaded into an unfamiliar location. Fecal samples were collected daily at approximately 11:00 for at least 3 days before and 14 days after transport (sampling details given in Table 1). Fecal samples were collected within 1 h after defecation and directly transferred to a −20 °C freezer.

### Effects of collection time

To assess the effect of time of day on FGM concentration, samples were collected from Giraffe 1 immediately following every defecation over a 48-h period. A total of 32 samples were collected beginning at 06:00 on 17 October 2012 and ending at 06:00 on 19 October 2012. Fecal samples were frozen within 1 h of collection.

### Sample processing and extraction procedures

Samples collected during the ACTH challenge trials as well as for determining effects of collection time were pulverized, mixed, and 0.1 - 0.11 g extracted by adding 3 ml 80 % methanol and vortexed for 15 min. Feces were then pelleted by centrifuging at 2000 g for 15 min and the supernatant decanted into polypropylene tubes for storage. For shipping or storage at room temperature, 0.5 ml of each fecal sample’s extract were transferred into Eppendorf tubes and air-dried [35]. Subsequent steroid analyses were conducted at the Wildlife Reproductive Centre, Taronga Conservation Society, Dubbo, NSW Australia.

Samples collected for monitoring the effect of transport were extracted by weighing 0.495–0.505 g of wet feces, adding 5 ml 80 % methanol, vortexing for 30 min, and centrifuging at 2500 g for 15 min [35]. Sample processing and assays were performed at the University of Veterinary Medicine, Vienna, Austria.

### Assay procedures

Fecal extracts resulting from the ACTH challenge test samples were measured for immunoreactive FGM concentrations using six different enzyme immunoassays (EIAs), namely a cortisol, corticosterone, 11-oxoetiocholanolone I (detecting 11,17-dioxoandrostanes; 11,17-DOA), 11-oxoetiocholanolone II (detecting FGM with a 5β-3α-ol-11-one structure; 3α,11-oxo-CM), 5α-pregnane-3β,11β,21-triol-20-one (measuring 3β,11β-diol-CM), and an 11β-hydroxyetiocholanolone EIA (measuring 3α,11β-diol-CM). Detailed assay characteristics, including references providing full descriptions of the assay components and cross-reactivities, are provided in Table 2. Samples collected for monitoring the effect of transport and collection time were assessed using only the two 11-oxoetiocholanolone EIAs, which most effectively identified FGM peaks following ACTH administration (Fig. 2).

**Table 2:** Details of the enzyme immunoassays (EIAs) used

EIA (source laboratory, abbrev.)	Sensitivity (ng/g feces)	Intra-assay CV^a^	Inter-assay CV^b^	References
Cortisol (Munro, R4866)	2.3	4.9 %	3.7 %, *n* = 4	[77, 78]
Corticosterone (Munro, CJM06)	17.6	8.1 %	8.1 %, *n* = 4	[79, 80]
11-oxoetiocholanolone I (Palme, 72a, 11,17-DOA)	7.2	4.8 %	12.3 %, *n* = 5	[63, 81]
11-oxoetiocholanolone II (Palme, 72T, 3α,11-oxo-CM)	9.6	8.8 %	13.8 %, *n* = 4	[61, 67]
5α-pregnane-3β,11β,21-triol-20-one (Palme, 37e, 3β,11β-diol-CM)	9.6	6.1 %	9.5 %, *n* = 4	[34]
11ß-hydroxyetiocholanolone (Palme, 69a, 3α,11β-diol-CM)	48.0	9.1 %	9.7 %, *n* = 4	[54, 82]

^a^Intra-assay CV = SD/mean percent binding for 10 wells each of high- and low-concentrated pool samples, averaged across concentrations

^b^Inter-assay CV = SD/mean percent binding for standards (72T, 69a) or high- and low-concentrated pool samples (other assays), averaged across concentrations, n indicates number of plates used

**Fig. 1:**
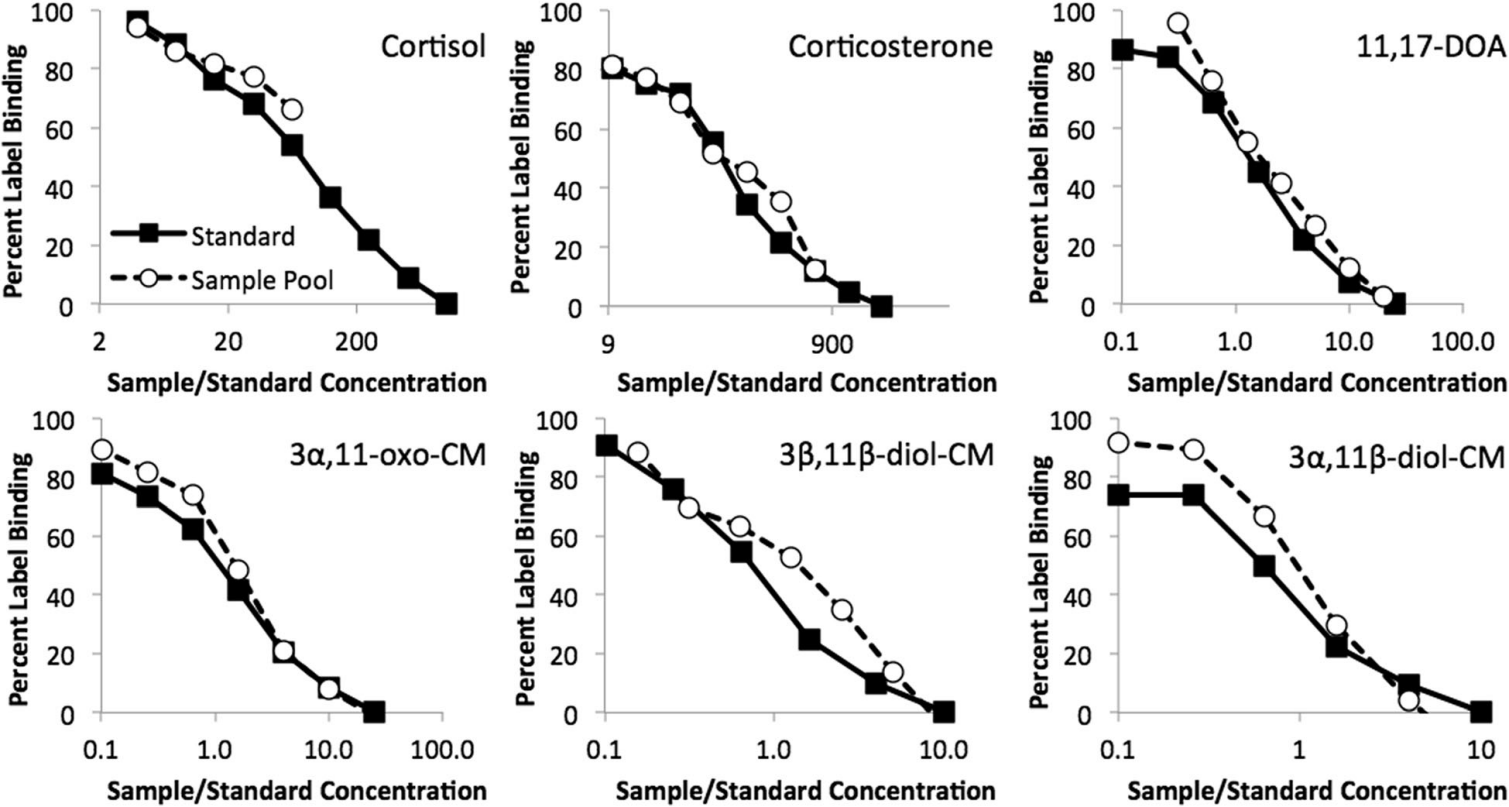
Parallelism of serial dilutions of a fecal extract pool with standards for each of the six EIAs. Correlations between percent binding for standards and samples within each assay were at least *r* = 0.96

To determine whether giraffe fecal extracts contained FGM that would bind to the antibody in each EIA in a way comparable to its standard, we first created a fecal extract pool consisting of equal volumes of 10 samples collected for the ACTH challenge experiment: 2 pre-injection, 2 at the expected peak (12–48 h post-injection), and 1 post-peak sample from each giraffe. For all assays, parallelism was then demonstrated by comparing serial dilutions of a fecal extract pool to serial dilutions of the steroid standard against which the antibody was raised [36]. Parallelism was acceptable for all assays but best for the two 11-oxoetiocholanolone assays; the correlation between percent binding for standards and samples within each assay was at least *r* = 0.96 (Mean_r_ = 0.98, Fig. 1). Using these data, appropriate dilution for samples in each assay was identified by determining what dilution produced close to 50 % sample binding for the pool.

### Data analysis

For data from both the ACTH challenges and the transport events, the range of baseline FGM for each individual as measured by each assay was determined by iteratively calculating a mean + 2SD threshold of concentration based on all samples collected from that individual [33, 37, 38]. At each step, samples that fell above the threshold were excluded and the mean and SD were recalculated until all remaining samples fell below the threshold and could be considered baseline values. After the baseline range was established, the post-injection peak was defined as the longest series of consecutive post-injection samples above the final mean + 2SD threshold. Data are available in Additional file 1.

Once the post-injection peak had been identified, three measures of assay effectiveness were computed. First, we found the duration of the peak by computing the difference between the times the first and last peak samples were collected. This allowed us to assess how useful the assay would be in identifying FGM peaks under field or zoo conditions with limited sampling frequency. Second, we calculated the fold difference by taking the concentration of the highest sample in the peak and dividing it by the mean concentration of the baseline samples. Third, we determined Z_peak_, the height of the peak as measured by the number of standard deviations away from the baseline mean, by taking the concentration of the highest sample in the peak, subtracting the mean concentration of the baseline samples, and dividing the result by the standard deviation of the baseline samples.

To assess the effects of time of day, we conducted a simple linear regression in which time since sunrise was used to predict FGM, and then a hierarchical linear regression in which time since sunrise was used to predict FGM after controlling for the day on which the samples were collected. Assays for which the addition of time since sunrise significantly increased R^2^ after the effect of collection day was accounted for were considered to detect a diurnal pattern. Data are available in Additional file 1 (see Giraffe 1).

## Results

### ACTH challenge test

**Fig. 2:**
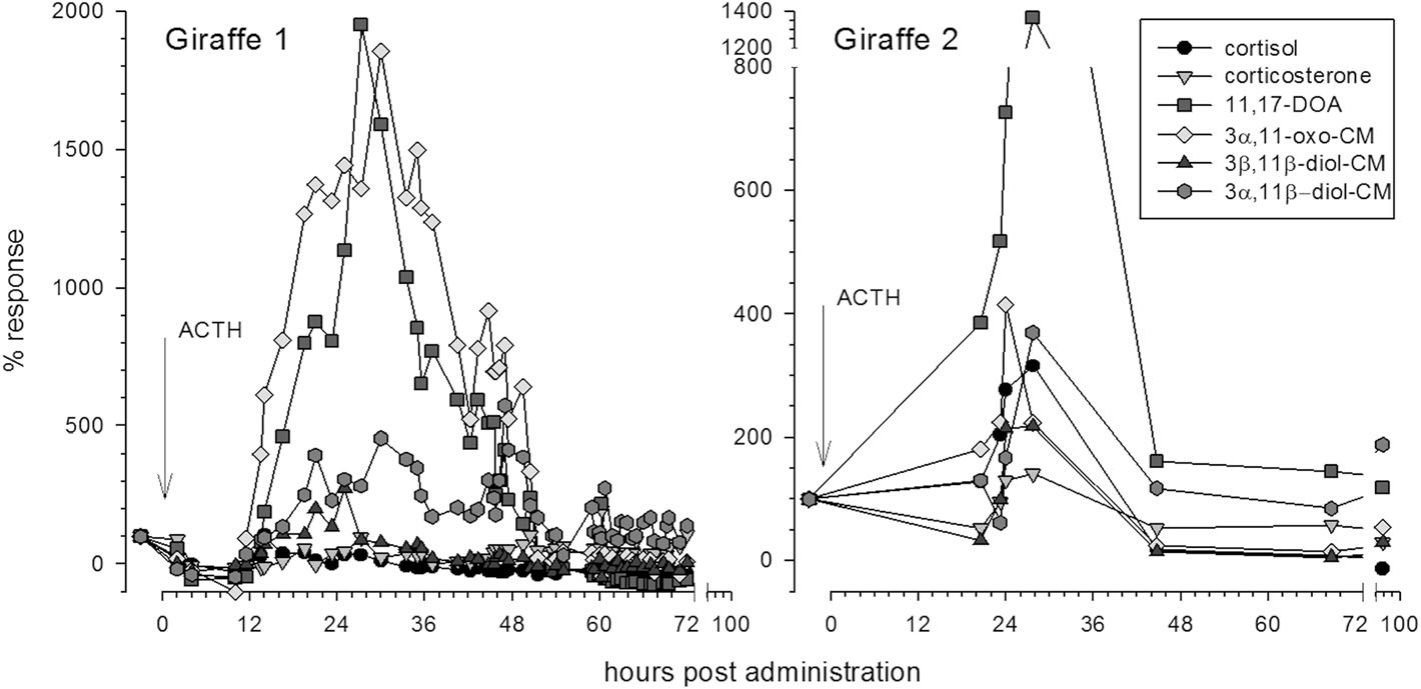
ACTH response of the two giraffes as measured by each of the six EIAs. Percent response was calculated by setting the individual median FGM value for pre-injection samples from each giraffe at 100 % and calculating the change in FGM levels for each post-injection sample. Vertical arrow marks injection time

All EIAs showed acceptable performance characteristics with giraffe samples (Table 2). Results of the assay comparison for ACTH samples are shown in Table 3 and Fig. 2. For both giraffes, the two antibodies raised against 11-oxoetiocholanolone (measuring 11,17-DOA and 3α,11-oxo-CM) performed best by all measures, identifying elevations in FGM levels above individual baseline for longer periods of time with respective peak values being comparatively higher than those determined by other assays (Fig. 2). The 3β,11β-diol-CM assay also performed acceptably for both individuals, though respective peak values were not as distinct from baseline as the ones determined by the 11-oxoetiocholanolone assays (Fig. 2). The cortisol, corticosterone, and 3α,11β-diol-CM EIAs detected peaks in Giraffe 1 but not Giraffe 2, and the peaks detected were substantially shorter in duration and differed less from baseline values (Table 3). One sample from Giraffe 2 was stored on ice for almost 2 h, which could have affected FGM [29, 39], but patterns of change over time were smooth, peaks detected in 11,17-DOA, 3α,11-oxo-CM, and 3β,11β-diol-CM included both samples held on ice and those frozen immediately, and this sample did not have the highest or lowest FGM for any assay.

**Table 3:** Assay comparison for the ACTH challenge tests

		Enzyme immunoassay targeting					
Animal	Measure	Cortisol	Cortico-sterone	11,17- DOA	3α,11-oxo-CM	3β,11β-diol-CM	3α,11β-diol-CM
Giraffe 1^a^	Latency to Peak	13.5 h	50.5 h	13.5 h	11.5 h	13.5 h	30.0 h
	Peak Duration (no. of peak samples)	6 h *n* = 4	<2 h *n* = 1	37 h *n* = 24	39 h *n* = 25	22 h *n* = 12	5 h *n* = 3
	Fold Increase (X_peak_/M_baseline_)	2.6	1.6	33.4	18.2	4.1	2.6
	Z _peak_	7.4 sd	2.2 sd	64.4 sd	79.6 sd	13.6 sd	3.4 sd
Giraffe 2^b^	Latency to Peak	no peak	no peak	20.5 h	24.0 h	23.2 h	no peak
	Peak Duration (no. of peak samples)			7 h *n* = 4	<5 h *n* = 1	5 h *n* = 3	
	Fold Increase (X_peak_/M_baseline_)			9.6	2.9	2.9	
	Z _peak_			17.1 sd	3.3 sd	11.3 sd	

^a^For Giraffe 1, fecal samples collected from every defecation from 12 h pre-injection to 72 h post-injection

^b^For Giraffe 2, fecal samples collected daily (between 8 am and 10 am) for 5 days before and 4 days after injection, as well as from every defecation between 20 h and 28 h post-injection

### Transport

**Fig. 3:**
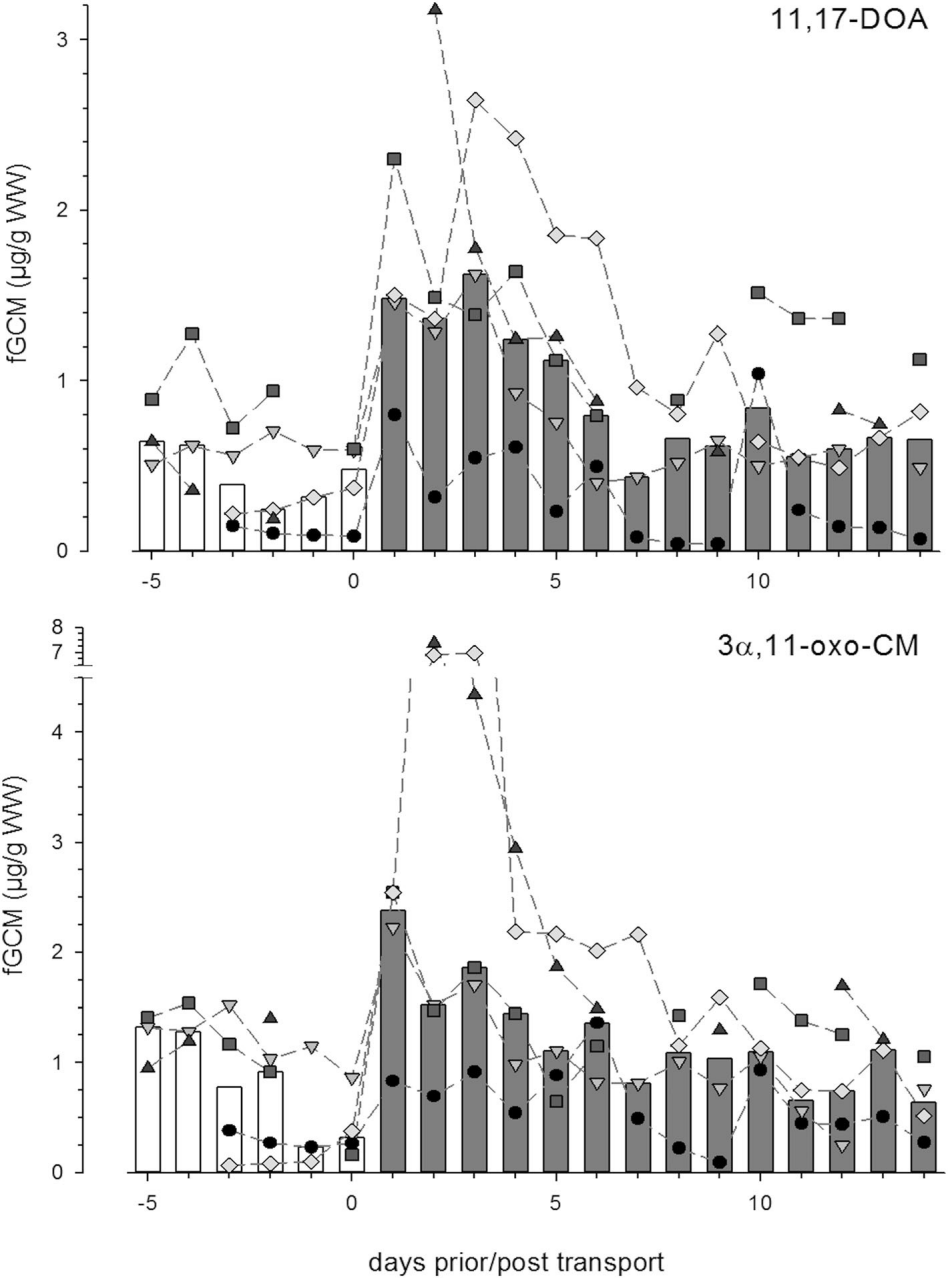
FGM responses of five giraffes to transport as measured by the 11,17-DOA and 3α,11-oxo-CM EIAs. Bars show median FGM values, points and lines show FGM concentrations for each individual. Both assays show significant peaks following transport for every individual (11,17-DOA peak: M_latency_ = 1.6 days, M_duration_ = 4.2 days, M_fold difference_ = 4.5x, M_Z_ = 10.7sd; 3α,11-oxo-CM peak: M_latency_ = 2.4 days, M_duration_ = 2.4 days, M_fold difference_ = 3.8x, M_Z_ = 7.4sd)

As samples from the transported giraffes were collected once per day and only the two 11-oxoetiocholanolone EIAs identified peaks lasting more than 24 h following ACTH challenge, we selected these two EIAs to assess the influence of transportation on FGM levels. Post-transport peaks were identified in both 11,17-DOA and 3α,11-oxo-CM in all 5 giraffes tested, regardless of the sex of the giraffe (Fig. 3).

The responses of individual giraffe as measured by both 11-oxoetiocholanolone EIAs are presented in Table 4. Three of the five giraffes showed peak 11,17-DOA concentrations the day after transport and the longest delay to peak values was 2 days. Peak duration averaged 4.2 days (range: 2–6 days) and intensity was comparable to the ACTH challenge (Fold difference: Median = 5.99, range: 2.33–8.00; Z_peak_: Median = 12.24, range: 4.74–20.10). As in the ACTH challenge, 11,17-DOA varied less within the baseline than 3α,11-oxo-CM. Peak 3α,11-oxo-CM occurred at a longer delay; only two of the five giraffes showed peaks the day after transport and the longest delay to peak was 6 days. Peaks in 3α,11-oxo-CM were less prolonged (Mean = 2.4 days, range: 1–3 days) but of similar overall intensity (Fold difference: Median = 2.78, range: 2.48–5.19; Z_peak_: Median = 4.37, range: 3.26–16.87). The two juvenile females (Giraffes 4 and 5) had exceptionally high 3α,11-oxo-CM concentrations in the three days following transport.

**Table 4:** Individual giraffe’s responses to transport as measured by 11-oxoetiocholanolone EIAs

EIA	Measure	Giraffe 3	Giraffe 4	Giraffe 5	Giraffe 6	Giraffe 7
11-oxoetiocholanolone I, targets 11,17- DOA	Latency to Peak	1 d	2 d	2 d	1 d	1 d
	Peak Duration	6 d	4 d	2 d	4 d	5 d
	Fold Increase (X_peak_/M_baseline_)	8.00	6.00	5.99	2.33	3.11
	Z _peak_	20.10	12.24	6.88	4.74	13.14
11-oxoetiocholanolone II, targets 3α,11-oxo-CM	Latency to Peak	6 d	2 d	3 d	1 d	1 d
	Peak Duration	1 d	3 d	2 d	3 d	3 d
	Fold Increase (X_peak_/M_baseline_)	2.78	5.19	3.04	2.48	2.66
	Z _peak_	3.62	16.87	3.26	4.37	5.42

### Effects of collection time

**Fig. 4:**
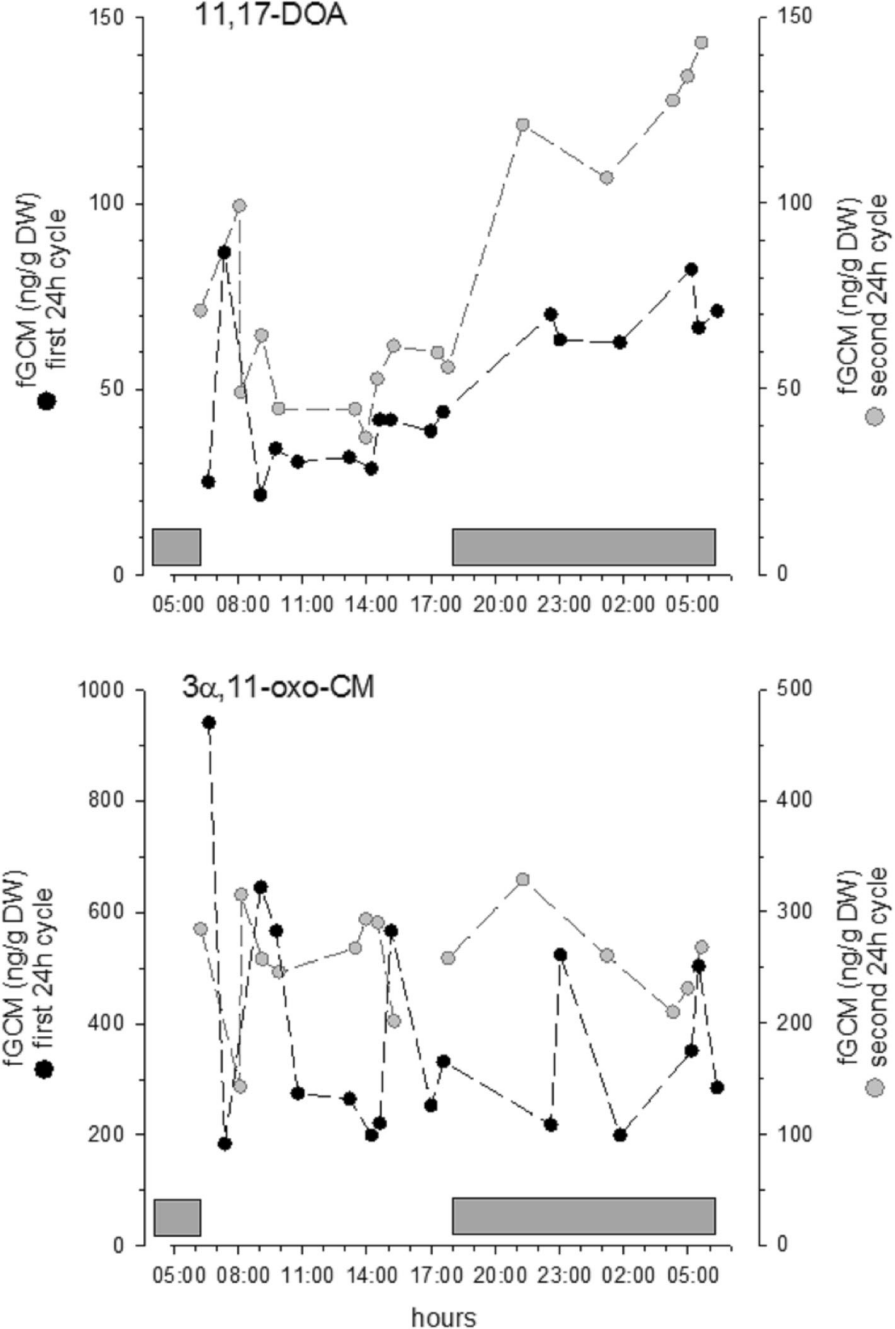
24 h patterns in baseline FGM levels in one male captive giraffe. 11,17-DOA (*top*) 3α,11-oxo-CM (*bottom*)

11,17-DOA and 3α,11-oxo-CM were affected by collection time. With the 11,17-DOA EIA, time since sunrise significantly predicted higher FGM concentrations (*R* ^2^ = 0.38, F_1,30_ = 18.05, *p* < 0.001, β = 0.61, t = 4.25, Fig. 4), and this relationship was still significant even when collection day was controlled (ΔR^2^ = 0.37, F_1,29_ = 26.49, *p* < 0.001, β = 0.61, t = 5.15). 3α,11-oxo-CM revealed a pulsatile excretion pattern, but it was not significantly predicted by time since sunrise (without controlling collection day: *p* = 0.364; controlling collection day: *p* = 0.345, Fig. 4).

## Discussion

### Assay selection

We demonstrated that FGM changes produced by both physiological stimulation (ACTH) and an environmental event (transport) could be reliably measured in daily fecal samples from giraffes using two 11-oxoetiocholanolone EIAs (measuring FGM with a 3α,11-oxo and 11,17-DOA structure, respectively). 11-oxoetiocholanolone EIAs best identified FGM peaks under field or zoo conditions where sampling frequency is limited. These assays found more samples with significant FGM elevation, better discriminated peak samples from baseline samples, identified a transport response with once-daily sampling, and were effective for both males and females. The other four EIAs we tested detected an increase in FGM following the ACTH challenge in at least one animal, but did not provide as clear and reliable peak detection and so were not as sensitive or consistent as the 11-oxoetiocholanolone EIAs.

For the ACTH challenge with more frequent sampling and larger dose (Giraffe 1: 1 IU/kg), both 11-oxoetiocholanolone EIAs had peaks of similar duration and intensity as measured by fold difference. However, the Z_peak_ for 3α,11-oxo-CM was larger because there was less variability among baseline samples. With less frequent sampling and lower ACTH dose (Giraffe 2: 0.7 IU/kg), peak duration and intensity were reduced for both assays; in fact, the peak in 3α,11-oxo-CM was identified in only a single sample. We did not collect blood samples following ACTH administration to verify an increase in serum GC because it would have required anesthetizing the giraffe. We therefore cannot rule out the possibility that Giraffe 2’s ACTH challenge did not sufficiently increase circulating GC, resulting in the failure of the Cortisol, Corticosterone, and 11ß-hydroxyetiocholanolone EIAs to detect a FGM peak. However, given that the other three assays did detect a peak it is unlikely the ACTH challenge failed entirely. ACTH injections produce a dose-dependent increase in GC that affects both serum GC concentration and clearance time [40, 41]. As the dose of ACTH used in these challenges was on the low end of the range used in other published challenges (1 and 0.7 IU/kg here; 0.5 to 12.5 IU/kg in [42]; 0.5 to 31 IU/kg in [43]), and ACTH was administered over a span of less than 2 min, the resulting increase in GC would be expected to be of shorter duration and perhaps lower intensity than those seen in other ACTH challenge studies. In contrast to the acute GC increase produced by the ACTH challenge, transport to a new environment is a longer-duration, more substantial environmental stressor [44]. Under these conditions, both 11-oxoetiocholanolone EIAs reliably detected a peak in all animals, but 11,17-DOA peaks had more rapid onset and greater duration.

The 11-oxoetiocholanolone I and II EIAs appear to robustly measure giraffe FGM. Our experiments relied on opportunistic access to zoo-housed giraffes, so there was considerable variability in the management of and procedures applied to each individual animal. We noted possible effects of differences in ACTH dose and sampling frequency above. In addition, the two animals subjected to ACTH challenges also differed in age, housing, climate, diet, and time of ACTH administration. While these factors likely increased variability in FGM patterns between the individuals [21, 29, 36, 45, 46], three assays (measuring 3α,11-oxo-CM, 11,17-DOA, and 3β,11β-diol-CM) reliably detected post-injection FGM increases despite these differences. Similarly, demographic factors, management, season of transport, and length of transport differed among giraffes in the transport study. This study also used a somewhat different extraction protocol than the ACTH challenge. Despite these differences, the 11-oxoetiocholanolone I and II EIAs still detected significant FGM increases as a result of transport in every animal. We noted differences in the shape of the FGM response to transport among individuals, which could be explained by differences in demographic effects, management, season, transport variables, or individual differences in reactivity to the same event [47]. The contributions of each of these factors to variation in FGM in giraffes should be studied in more detail. In addition, published experiments of domestic ruminant feces stored at room temperature suggest results of the 11-oxoetiocholanolone EIAs may be prone to changes during storage [48, 49]; these effects should be evaluated for exotic ruminants.

11,17-DOA also showed diurnal variation in giraffe feces similar to those found in African buffalo (*Syncerus caffer*) [45]. With only a single subject and no serum hormone data, it is not possible to determine whether the observed pattern reflects a circadian rhythm in circulating hormones; blood samples have shown circadian secretion rhythms for cortisol are present in some ruminants [50] but are weak [51, 52] or absent [53] in others. The diurnal variations in 11,17-DOA may be idiosyncratic to this individual or to the days samples were collected. However, the more substantial 11,17-DOA peaks in response to both ACTH and transport and a consistent diurnal pattern suggests that the 11-oxoetiocholanolone I EIA is the most biologically sensitive assay we tested for giraffes.

Though the 11-oxoetiocholanolone I EIA (for 11,17-DOA) is more sensitive, there may be situations where the 11-oxoetiocholanolone II EIA (for 3α,11-oxo-CM) is still preferable. First, for the two juvenile female giraffes, the 11-oxoetiocholanolone II EIA detected a greater change in FGM following transport than the 11-oxoetiocholanolone I EIA. Cross-reactivity with gonadal hormones could be responsible for the difference [54]. However, cross-reactivity should increase FGM concentrations independent of the transport event, and these two individuals only had higher FGM concentrations in the post-transfer peak, not in baseline. Instead, immunoreactive 3α,11-oxo-CM may be more prevalent in the metabolite profiles of these two giraffes, perhaps as a result of age- of sex-specific differences in metabolism [34, 55]. Our data support using 3α,11-oxo-CM in studies of juvenile female giraffes. Second, we found systematic changes in 11,17-DOA concentrations across time of day, so studies using this assay must control for the time of day during either sampling or statistical analysis. If such control is not possible, assessing 3α,11-oxo-CM using the 11-oxoetiocholanolone II EIA is advised. Finally, if the aim of one’s study is to identify husbandry or environmental events that are perceived as very stressful, as in [56, 57], the less biologically sensitive 11-oxoetiocholanolone II EIA may set a higher criterion for identifying peaks and so identify a smaller number of events.

### Ruminant FGM measurement

Consistent with other studies of ruminants, we found that group-specific assays designed to target GC metabolites were more effective at measuring FGM than assays using antibodies raised against circulating GC. Circulating GC are metabolized extensively in the liver and additionally by gut microbes before excretion, so the types and ratios of specific FGM differ across sex and species (for review, see [21]). While cortisol or corticosterone EIAs effectively measure FGM in many species including some ruminants [42, 58–60], native GC are absent from the feces of most vertebrates [21, 42, 61] so this measurement depends on having a sufficient concentration of FGM that have retained the characteristic of GC structure recognized by the particular antibody. A radiometabolism study could be used to identify exactly which FGM are present in a particular species and allow targeting of assays based on metabolite profiles [61, 62], but it is difficult to conduct such a study in wildlife species the size of a giraffe. Instead, comparing assays that target different metabolite structures can be used to infer some information about metabolite profiles [43].

So-called group-specific assays targeting GC metabolites [32] have been effectively used to measure FGM in ruminant species for which GC-specific assays have failed (e.g., sheep, cattle [63, 64]). Our study adds giraffes to the list of ruminant species for which one of the 11-oxoetiocholanolone EIAs is most effective in measuring FGM (dromedary camels: *Camelus dromedarius* [65], cattle: *Bos primigenius f. taurus* [64], sheep: *Ovis ammon f. aries* [61, 64], goats: *Capra aegagrus f. hircus* [66]*,* red deer: *Cervus elaphus* [67], roe deer: *Capreolus capreolus* [68], pampas deer: *Ozotoceros bezoarticus* [69], llama: *Lama guanicoe f. glama*, alpaca: *Vicugna vicugna f. pacos*, and vicuña: *Vicugna vicugna* [70]). The success of the 11-oxoetiocholanolone EIAs in such a large number of ruminant species suggests 5β-reduction may be a common metabolic pathway for GC in ruminants. However, all assays we used detected some post-event FGM peaks, which may indicate giraffes excrete a great variety of measurable metabolites like other ruminants. While FGM assays should still be validated for each species, we suggest FGM validation studies in other ruminants should begin by trying a group-specific 11-oxoetiocholanolone EIA.

### Applications for giraffe health, welfare, and conservation

Our validation of multiple assays to measure FGM in giraffes provides a tool that can be applied in monitoring health and welfare, as well as conservation research. Zoos are particularly interested in generating data that will allow them to assess and improve giraffe health and management. The adrenal system responds similarly to exciting and stressful events, so FGM changes are best interpreted in the context of information about the animal’s environment and behavior [22]. Adding FGM measurement to behavior data in other species has allowed animal managers unique insights into animals’ perceptions of captive environments. For example, captive wombats showed behavioral habituation to interaction with humans, but their FGM response to these interactions remained unchanged, suggesting they may have developed learned helplessness [56]. Physiological measures of giraffe health and perceived stress will help supplement behavioral welfare indicators and may reveal critical aspects of the relationship between captive management and welfare.

As giraffes can be individually identified by their natural markings [71], validation of FGM assays will also allow researchers to obtain longitudinal measures of adrenal activity from individual wild giraffes. Non-invasive longitudinal adrenal assessment will aid in determining the effects of environmental degradation and conservation efforts on giraffe health, reproduction, diet, and life history strategies [31, 72]. Giraffes in captivity might reasonably be expected to have different baseline levels of GC and/or respond differently to stressors than wild giraffes [72], so field studies should seek within-individual patterns of FGM that can be attributed to particular events. Recent studies have used FGM to evaluate how primates perceive conservation-related stressors, from ecotourism (orangutans: *Pongo pygmaeus morio* [73], gorillas: *Gorilla gorilla gorilla* [74]) to long-term changes in food distribution and disease prevalence (red colobus: *Procolobus rufomitratus* [75]); similar studies could now be undertaken in giraffes. Across species, Dantzer and colleagues’ [76] meta-analysis found that anthropogenic disturbances are consistently associated with increased FGM, but argue that these FGM changes may enhance survival in disturbed populations. However, there are well-described negative health consequences of chronic exposure to elevated GC [22], so the relationship between FGM and fitness in disturbed populations is ripe for research. Giraffes live in a diverse array of habitats with varying degrees of human disturbance; in some areas giraffe populations are increasing, while in most of the range populations are crashing. Giraffes are therefore an ideal species in which to use FGM measures to investigate questions of captive health, welfare, physiology, ecology, and conservation.

## Conclusions

We conclude the assay for 11,17-dioxoandrostanes is the most sensitive assay tested for FGM in giraffes and the assay for FGM with a 5β-3α-ol-11-one structure is also effective. 11-oxoetiocholanolone enzyme immunoassays have now been demonstrated to be successful in a wide variety of ruminant species, providing indirect evidence that 5β-reduction may be a common metabolic pathway for glucocorticoids in ruminants. As FGM peaks were detected in at least some giraffes using all assays tested, giraffes appear to excrete a wide variety of different FGM. The assays validated here will provide a valuable tool for research on the health, welfare, and conservation of giraffes.

## Additional file

Additional file 1: Full dataset in Microsoft Excel workbook format. (XLSX 61 kb)

## Abbreviations

ACTH: Adrenocorticotropic hormone
EIAs: Enzyme immunoassays
FGM: Fecal glucocorticoid metabolites
GC: Glucocorticoids
HPA: Hypothalamic-pituitary-adrenal
IUCN: International Union for Conservation of Nature
NZG: National Zoological Gardens of South Africa
